# Efficient and Privacy-Preserving Energy Trading on Blockchain Using Dual Binary Encoding for Inner Product Encryption †

**DOI:** 10.3390/s21062024

**Published:** 2021-03-12

**Authors:** Turabek Gaybullaev, Hee-Yong Kwon, Taesic Kim, Mun-Kyu Lee

**Affiliations:** 1 Department of Electrical and Computer Engineering, Inha University, Incheon 22212, Korea; turabek.gaybullaev@gmail.com (T.G.); heeyong.kr@gmail.com (H.-Y.K.); 2 Department of Electrical Engineering and Computer Science, Texas A&M University, Kingsville, TX 78363, USA; taesic.kim@tamuk.edu

**Keywords:** integer comparison, inner product, functional encryption, blockchain, energy trading

## Abstract

The rapidly increasing expansion of distributed energy resources (DER), such as renewable energy systems and energy storage systems into the electric power system and the integration of advanced information and communication technologies enable DER owners to participate in the electricity market for grid services. For more efficient and reliable power system operation, the concept of peer-to-peer (P2P) energy trading has recently been proposed. The adoption of blockchain technology in P2P energy trading has been considered to be the most promising solution enabling secure smart contracts between prosumers and users. However, privacy concerns arise because the sensitive data and transaction records of the participants, i.e., the prosumers and the distribution system operator (DSO), become available to the blockchain nodes. Many efforts have been made to resolve this issue. A recent breakthrough in a P2P energy trading system on an Ethereum blockchain is that all bid values are encrypted using functional encryption and peer matching for trading is performed securely on these encrypted bids. Their protocol is based on a method that encodes integers to vectors and an algorithm that securely compares the ciphertexts of these vectors. However, the comparison method is not very efficient in terms of the range of possible bid values because the amount of computation grows linearly according to the size of this range. This paper addresses this challenge by proposing a new bid encoding algorithm called dual binary encoding, which dramatically reduces the amount of computation as it is only proportional to the square of the logarithm of the size of the encoding range. Moreover, we propose a practical mechanism for rebidding the remaining amount caused when the amounts from the two matching peers are not equal. Finally, the feasibility of the proposed method is evaluated by using a virtual energy trade testbed and a private Ethereum blockchain platform.

## 1. Introduction

The issue of critical shortage and depletion of natural resources worldwide has been one of the most significant discussions in the energy sector [1]. Meanwhile, the consequences of climate change have global effects on every region of the world, but the distribution of impacts is likely to be inherently unequal [2]. It affects more seriously the developing countries and especially the poor, as they have the least economic, institutional, scientific, and technical capacity to adapt [1,2]. To cope with these issues, there have been many global activities to achieve inclusive and sustainable development, including the United Nation (UN)’s long-term agenda for Sustainable Development Goals [3] which replaced the previous Millennium Development Goals, and the UN Development Programme’s Social and Environmental Standards [4]. These activities are also transforming business, corporate governance, and corporate social responsibility models, by ensuring that limited resources are used responsibly and efficiently [1]. In this context, comprehensive reforms to the energy sector are also necessary. The International Energy Agency (IEA) recommends promoting market-oriented energy sector reform, improving energy security and diversification, and increasing and reinforcing measures for environmental protection, especially emissions reduction [5]. Renewable energy has a key role in these reforms.

The legacy power grids were designed to send energy in one way from the generator to the consumers. However, current electric power grids are undergoing a rapid transition because of the increasing expansion of distributed energy resources (DER), such as renewable energy systems (RES), energy storage systems, electric vehicles, and controllable loads in subtransmission systems (i.e., large-scale DER) and distribution systems (i.e., middle- or small-scale DER) [6]. For example, households with a photovoltaic system can generate electricity as a small-scale DER, whereas a large wind farm connected to subtransmission systems is a large-scale DER. At the same time, advanced information and communication technologies (ICT) such as the Internet of Things (IoT), cloud computing, and 5G technology adopted for power systems and DER have provided advanced metering and real-time operational tools, allowing dynamic automated management of DER at large scale [7]. Presently, DER provides grid services such as demand response, which usually changes power consumption to balance the power supply with the demand in distribution systems [8], mitigation of RES oversupplies [9], and fast grid recovery from blackouts (i.e., black start [6]). Therefore, DER owners can have opportunities to participate in electricity markets as prosumers for grid services managed by a distribution system operator (DSO). Furthermore, the concept of peer-to-peer (P2P) energy trading has been proposed for further flexible and resilient local grid services under a more complex future power grid environment with prosumers, decentralized energy systems, and new generation and consumption patterns, as well as for increasing the benefits to DER owners [10]. This localized P2P trading is the most recent trend regarding the energy industry and adopting inclusive sustainable models. Recently, blockchain has emerged as the best method for P2P energy trading because of the available blockchain technologies, including a combination of trust mechanisms among participants, such as distributed ledger, consensus algorithm, and smart contracts  [11].

According to a recent online survey experiment conducted by [11], respondents favor established authorities, e.g., a DSO, as a trading organizer, since energy trading requires numerous safety measures. Simultaneously, blockchain has been considered an attractive solution for peer matching and trading because of the transparency feature of blockchain transactions [12]. Therefore, we consider a hybrid approach, i.e., even when trading participants agree on an energy trade over a blockchain, they still need to use the power system infrastructure managed by a DSO to physically exchange power, thus requiring the DSO to participate in a blockchain network. In this setup, however, privacy concerns arise from potential P2P participants and the DSO because sensitive data and transaction records are accessible by the blockchain nodes [11]. There have been many efforts to resolve the privacy issue in P2P energy trading. Recently, Son et al. [13] proposed the use of an Ethereum blockchain for P2P energy trading, which enables fair peer matching and provides the privacy of matching details through encryption of bids. Privacy-preserving peer matching is maintained by encrypting the bid values from peers using functional encryption (FE) and by performing FE-based smart contracts on encrypted bids. Furthermore, when matching is successful, the matching peers are not allowed to discard their bids, i.e., non-repudiation for the bids is provided. In [13], the authors constructed a prototype of an energy trading system consisting of smart meters, Ethereum blockchain, and DSO server, which demonstrated the feasibility of the proposed solution. The novel functionality of FE in [13] was secure integer comparison without additional interaction of the parties [13]. However, the integer comparison method in [13] was not very efficient in terms of the range of possible bid values because the amount of computation grows linearly in the cardinality of the set of possible bid values. This may make the system very inefficient, especially when a greater range of bid values is to be supported.

In this paper, we introduce a new bid encoding algorithm called the dual binary encoding algorithm. This new encoding method is used in combination with FE to provide an efficient and secure integer comparison using multiple inner products of vectors that encode bid values. With the new encoding method, the vector dimension of an encoded bid value, and, hence, the amount of computation, grows with the square of logarithm of the bid range, which is in stark contrast to the linear growth in [13]. In the experimental result, the proposed algorithm showed a noticeable reduction in the computation time of encoding a bid value as well as in the gas cost reduction for the blockchain operations thanks to fewer vector elements than those in the previous work [13]. We also provide a rebidding function for the remaining amount of power after two bids are matched, which was not possible in [13]. We conducted a field test to verify the feasibility and practicality of the proposed solution.

## 2. Background and Related Work

### 2.1. Energy Blockchain

A blockchain is a distributed and immutable ledger that ensures integrity and transparency through chronologically ordered and cryptographically signed data blocks [14]. The idea of the blockchain was initially developed from Bitcoin [15]; however, there was a limitation due to script language functionality. Ethereum [16] is the most widely used protocol in blockchain, which by supporting Turing-complete programming languages, enables the implementation of complex programs [17,18]. Among the many blockchain-related research results, blockchain applications for P2P energy transactions have attracted the attention of a growing number of researchers studying blockchain in the energy field [19].

The adoption of blockchain in energy trading started with the experiment in Brooklyn, NY, USA, where solar power was sold from households directly to different households [20]. Blockchain is well suited for decentralized energy sectors, and, therefore, the implementation of blockchain technologies for P2P energy trading is being widely investigated. For example, Sabounchi et al. [21] proposed a model for P2P electricity trading between prosumers in residential microgrids. Blockchain-based local energy trading with home energy management and the demurrage mechanism was proposed in [22], and other state-of-the-art works are reviewed in [23]. The most recognized projects using blockchain for energy trading are Power Ledger [24], which is a platform for energy trading, and LO3 Energy’s Brooklyn Microgrid [25,26], which is an energy marketplace. There are also other projects, such as SolarCoin [27]. However, none of the above-mentioned projects tackled privacy issues. It is well known that many systems and techniques proposed for blockchains have issues with privacy and anonymity [28,29,30]. Therefore, a great number of studies have been conducted to resolve these issues for various blockchain applications. For example, Stamatellis et al. [31] proposed a solution for privacy-preserving healthcare framework on a blockchain, which provides anonymity and unlinkability. Prada-Delgado et al. [32] and Asif et al. [33] provided hybrid solutions that use blockchains combined with physical unclonable functions (PUFs) for IoT and IoE, respectively, where methods for clone-proof device identification and authentication using blockchains were proposed. Zerocash [34] and Zether [35] are well-known confidential cryptocurrency transfer mechanisms that ensure user anonymity. There are more general solutions using zero-knowledge proofs including zk-SNARK [36] and BulletProof [37]. However, most of the previous approaches aimed at keeping a type of footprint or proof on the blockchain, but none of the above-mentioned solutions were designed for directly performing confidential transactions on encrypted data on the blockchain. In the literature on energy blockchains, we can also find various proposals to provide privacy for P2P energy trading. Pseudonym-based solutions on consortium-based blockchains [38,39] and similar token-based energy trading, where peers are anonymous, were proposed [12]. To prevent malicious data mining and linking threats, the authors in [40] proposed a technique that adds random noise to the distribution of trade. In addition, there was an effort to achieve a secure trading mode on a cross-chain trading platform for multi-microgrid systems by providing key management and interoperability protocols [41]. Moreover, secure search schemes over encrypted data on blockchain for e-commerce and electronic health records were proposed in [42,43] However, the only previous work in the literature that allows direct trading transactions on encrypted data was [13]. In [13], privacy-preserving matching protocol on an Ethereum-based energy blockchain was introduced, where both the bid values and the identities of users are kept secret from other users.

### 2.2. FHIPE and Integer Comparison

The functional encryption scheme [44] used in this work is constructed using a cryptographic pairing. Let $G_{1}$ and $G_{2}$ be two additive groups, and let $G_{T}$ be a multiplicative group. However, for notational convenience, we also formulate the group operations in $G_{1}$ and $G_{2}$ multiplicatively. A cryptographic pairing is defined as a map $e:G_{1}\times G_{2}\to G_{T}$ that satisfies the following properties:

- The map *e* and the group operations in $G_{1},G_{2}$, and $G_{T}$ can be efficiently computed.
- The map *e* is bilinear, such that, for all $x,y\in Z_{q}$, the map *e* satisfies $e\left(P^{x},Q^{y}\right)=e{\left(P,Q\right)}^{xy}$, where *q* is the order of $G_{1}$ and $G_{2}$, and $P\in G_{1},Q\in G_{2}$.
- The map *e* has non-degeneracy, i.e., e(P,Q)≠1 if *P* and *Q* are not the identity elements in $G_{1}$ and $G_{2}$, respectively.

For any group element t∈G and a row vector $v=\left(v_{1},\ldots ,v_{n}\right)\in Z_{q}^{n}$, where G is a group of prime order *q* and n∈N, we use $t^{v}$ to denote a vector of group elements $\left(t^{v_{1}},\ldots ,t^{v_{n}}\right)\in G^{n}$. The pairing operation over $G_{1},G_{2}$ is extended to vectors as follows:

(1) $$e\left(P^{v},Q^{w}\right)=\prod_{i=1,\ldots ,n}e\left(P^{v_{i}},Q^{w_{i}}\right)=e{\left(P,Q\right)}^{\langle v,w\rangle },$$

where $v=\left(v_{1},\ldots ,v_{n}\right)\in Z_{q}^{n}$, $w=\left(w_{1},\ldots ,w_{n}\right)\in Z_{q}^{n}$, and 〈v,w〉 is the inner product of v and w.

We use a special case of functional encryption (FE), namely function-hiding inner product encryption (FHIPE) proposed by Kim et al. in 2018 [44]. FE is an encryption scheme that performs operations on encrypted data, producing the result as a decrypted value [45,46,47]. FHIPE is a special type of FE, where its secret key and ciphertext are associated with vectors [44,48,49,50,51]. Let a and b be two vectors, and we denote their encryption by E(a) and E(b), respectively. The decryption operation can be carried out by any party taking two ciphertexts E(a) and E(b) as inputs. The result of this operation is 〈a,b〉, but no information other than this inner product is revealed about either **a** or **b**.

The original definition of the inner product encryption (IPE) schemes use four probabilistic polynomial time (PPT) algorithms: Setup, KeyGen, Encrypt, and Decrypt [44,48,49,50,51]. However, for many applications, it is more intuitive to denote KeyGen as *Left Encrypt* and Encrypt as *Right Encrypt*, as mentioned in [44]. We follow the definitions in [44] and define four PPT algorithms as follows, where ${\mathrm{Enc}}_{L}$ and ${\mathrm{Enc}}_{R}$ represent Left Encrypt and Right Encrypt, respectively:

- Setup($1^{\lambda }$): When a security parameter λ is given, the setup algorithm samples $G_{1}$, $G_{2}$, and $G_{T}$, and defines *e*. The generators $P\in G_{1}$ and $Q\in G_{2}$ are also selected. Then, it samples B from a general linear group of (n×n) square matrices whose elements are selected from $Z_{q}$, and computes the matrix $B^{*}=\det\left(B\right)\cdot {\left(B^{-1}\right)}^{T}$, where det denotes the determinant of a matrix. Finally, the setup algorithm outputs the public parameters $\mathrm{op}=(G_{1},G_{2},G_{T},q,e)$ and the secret key $\mathrm{sk}=(\mathrm{op},P,Q,B,B^{*})$, where *q* is the order of $G_{1}$ and $G_{2}$.
- ${\mathrm{Enc}}_{L}\left(\mathrm{sk},\alpha ,x\right)$: When the secret key sk, a random element $\alpha \in Z_{q}$, and a row vector $x=(x_{1},\ldots ,x_{n})$ are given, the left encryption algorithm outputs $\left(L_{1},L_{2}\right)=\left(P^{\alpha \cdot \det(B)},P^{\alpha \cdot x\cdot B}\right),$ where $L_{1}\in G_{1}$ and $L_{2}\in G_{1}^{n}$.
- ${\mathrm{Enc}}_{R}$(sk,β,y): When the secret key sk, a random element $\beta \in Z_{q}$, and a row vector $y=(y_{1},\ldots ,y_{n})$ are given, the right encryption algorithm outputs $\left(R_{1},R_{2}\right)=\left(Q^{\beta },Q^{\beta \cdot y\cdot B^{*}}\right),$ where $R_{1}\in G_{2}$ and $R_{2}\in G_{2}^{n}$.
- $\mathrm{Dec}(\mathrm{op},E_{L}\left(x\right),E_{R}\left(y\right))$: When the public parameters op and two ciphertexts $E_{L}\left(x\right)=\left(L_{1},L_{2}\right)$, $E_{R}\left(y\right)=\left(R_{1},R_{2}\right)$ are given, the decryption algorithm calculates $D_{1}=e\left(L_{1},R_{1}\right)$ and $D_{2}=e\left(L_{2},R_{2}\right)$. Finally, it seeks a solution for the discrete logarithm problem ${\left(D_{1}\right)}^{z}=D_{2}$. In case *z* exists, the decryption algorithm outputs it, which is equal to the inner product of x and y, i.e., 〈x,y〉; otherwise, it outputs a symbol that implies that there is no valid *z*.

Now, we explain the vector encoding method for energy prices used in [13]. Let the price be one of the elements in an ordered set P⊂Z, where Z is the set of integers. The elements in *P* are sorted in increasing order and labeled as $p_{1},\ldots ,p_{\left|P\right|}$. For instance, if P={31,32,…,40}, then $p_{1}=31,p_{2}=32,\ldots ,p_{10}=40$. Let $index_{P}\left(p_{i}\right)$ be a function that returns the index of element $p_{i}$ in *P*, where 1≤i≤|P|. For example, $index_{p}\left(32\right)=2$. To use the FHIPE scheme [44] described earlier, the previous method [13] encodes the price value $pp_{U}\in P$ of the user *U* into two |P|-dimensional vectors, $U^{L}$ and $U^{R}$, called the left and right vectors, respectively. $U^{L}$ is encoded such that the elements in it with an index less than $index(pp_{U})$ are 0 and the other elements are equal to 1. On the other hand, $U^{R}$ is encoded using one-hot encoding. For instance, if $pp_{U}=36$, $U^{L}=\left(0,0,0,0,0,1,1,1,1,1\right)$ and $U^{R}=\left(0,0,0,0,0,1,0,0,0,0\right)$. When two users $U_{1}$ and $U_{2}$ submit their prices $pp_{U_{1}}$ and $pp_{U_{2}}$ as $\left(U_{1}^{L},U_{1}^{R}\right)$ and $\left(U_{2}^{L},U_{2}^{R}\right)$, respectively, the inner product operation is performed to compare $pp_{U_{1}}$ and $pp_{U_{2}}$. To be exact, $\langle U_{1}^{L},U_{2}^{R}\rangle =1$ means $pp_{U_{1}}\leq pp_{U_{2}}$. As can be seen, the range of bid values was limited because the size of the vectors was linear in the range of elements, i.e., the number of distinct integers that can be represented.

## 3. Proposed Integer Comparison Method Using Dual Binary Encoding

To resolve the issue of the linear relation between the price range and the number of vector elements of the encoding method used in [13], we propose a new encoding algorithm that represents the same range of integer values with significantly fewer vector elements. This algorithm is a revised version of the method presented in the preliminary version of this paper [52], tailored for blockchains. As the execution time of IPE operations is almost proportional to the number of vector elements, the novel encoding algorithm noticeably improves the speed of the IPE.

**Figure 1:**
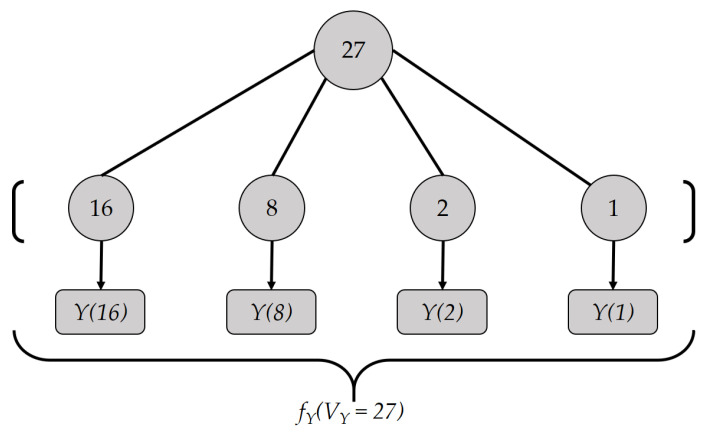
Encoding example: $f_{Y}\left(V_{Y}=27\right).$

Let $V_{X}$ and $V_{Y}$ be the two non-negative integers that will be compared. Both values, $V_{X}$ and $V_{Y}$, undergo different encoding processes denoted as $f_{X}$ and $f_{Y}$, respectively. Both encoding processes start by expressing the target integer as a sum of powers of 2 and then sorting them from the highest-order term. For example, if $V_{Y}=27$, then it is expressed as 27=16+8+2+1 and the terms 16, 8, 2, and 1 are encoded independently, as shown in Figure 1.

$$f_{Y}\left(V_{Y}=27=16+8+2+1\right)=\left[Y\left(16\right),Y\left(8\right),Y\left(2\right),Y\left(1\right)\right]$$

Similarly, encoding $f_{X}\left(V_{X}\right)$ is accompanied by partitioning $V_{X}$ as a sum of powers of 2 and encoding each term independently; however, this time each term is encoded twice as shown below:

$$f_{X}\left(V_{X}=27=16+8+2+1\right)=\left[X_{L}\left(16\right),X_{G}\left(16\right),X_{L}\left(8\right),X_{G}\left(8\right),X_{L}\left(2\right),X_{G}\left(2\right),X_{L}\left(1\right),X_{G}\left(1\right)\right]$$

**Figure 2:**
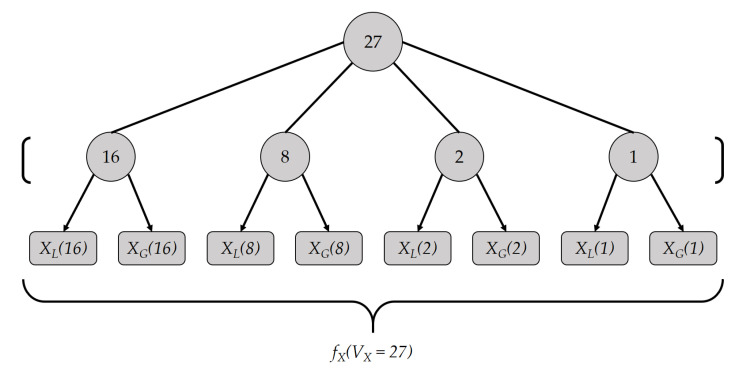
Encoding example: $f_{X}\left(V_{X}=27\right)$.

The encoding process $f_{X}\left(V_{X}\right)$ is also illustrated in Figure 2. $X_{L}$ and $X_{G}$ are two encoding methods, where the former is used to check the “less than or equal to” relation, whereas the latter is used to check the “greater than or equal to” relation. As we use two separate encoding methods $X_{L}$ and $X_{G}$, each of which resembles a binary representation, we name the proposed encoding method a *dual binary encoding*.

The remaining part of this section is structured in a bottom-up fashion. In Section 3.1, we explain how the individual terms in $V_{Y}$ are encoded using encoding the method *Y*. In Section 3.2, we explain how the method *Y* is used as a subroutine to construct the whole encoding process $f_{Y}$. In Section 3.3 and Section 3.4, we explain how the individual terms in $V_{X}$ are encoded using the methods $X_{L}$ and $X_{G}$. Section 3.5 shows how $X_{L}$ and $X_{G}$ are used as subroutines to construct the whole encoding process $f_{X}$. Section 3.6 shows how $V_{X}$ and $V_{Y}$ are compared with the help of $f_{X}$ and $f_{Y}$.

### 3.1. Subroutine Y

In this subsection, we describe the encoding method *Y* that is used in $f_{Y}$ as a subroutine to encode the individual terms of $V_{Y}$. In some cases, encoding for 0 may be required. Therefore, the input to *Y* is either 0 or a power of 2. Let *D* be an integer that satisfies D≥3. *Y* is a one-hot encoding method and outputs a *D*-dimensional vector for a predefined value *D*. The elements of an output vector are either 0 or 1. If D=8, it can encode the following values: { 0, $2^{0}$, $2^{1}$, $2^{2}$, $2^{3}$, $2^{4}$, $2^{5}$, $2^{6}$ }. To calculate all the elements of the vector, we compute the target index position *p*. If the input $V_{n}$ to *Y* is 0, then p=0. When $V_{n}=2^{i}$, the target index is p=i+1. Then, the *p*th vector element is set to 1, and all the other elements are set to 0. The detailed task of *Y* is depicted in Algorithm 1. For example, when $V_{n}=16$ and D=8, *Y* produces {0,0,0,0,0,1,0,0}:


Y(16,8)=
0124816326400000100

**Algorithm 1**$Y(V_{n},D)$: Subroutine to encode 0 or a power of 2
**Input**:Value to be encoded $V_{n}$, size of a vector *D***Ensure**:D≥3, $V_{n}$ is 0 or a power of 2 in the range $R\in [0,2^{D-2}]$**Output**:
*D*-dimensional vector with an encoded value $y=(y_{0},\ldots ,y_{D-1})$1:
**if** $V_{n}=0$ **then**
2: p←0 {*p*: Position of 1}3:
**else**
4:
$\;p\leftarrow \log_{2}V_{n}+1$
5:
**end if**
6:**for**i=0 to *D* **do** {0≤i<D}7: **if** i=p **then**8:
$\;\;y_{i}\leftarrow 1$
9: **else**
10:
$\;\;y_{i}\leftarrow 0$
11: **end if**
12:
**end for**
13:
**return** y



Table 1 presents all the possible values encoded with *Y* for D=8.

### 3.2. Encoding Method $f_{Y}$

By repeatedly using the subroutine *Y* to encode powers of 2, we can encode any integer. For example, when D=8, we obtain the following:

$$f_{Y}\left(V_{Y}=27=16+8+2+1\right)=\left[y_{0},y_{1},y_{2},y_{3}\right],$$

where

$$y_{0}=Y\left(16,8\right)=\left\{0,0,0,0,0,1,0,0\right\}$$

$$y_{1}=Y\left(8,8\right)=\left\{0,0,0,0,1,0,0,0\right\}$$

$$y_{2}=Y\left(2,8\right)=\left\{0,0,1,0,0,0,0,0\right\}$$

$$y_{3}=Y\left(1,8\right)=\left\{0,1,0,0,0,0,0,0\right\}$$

The range of $V_{Y}$ that can be expressed using eight-dimensional vectors ranges from 0 to 127=64+32+16+8+4+2+1. Generally, the range of integers that can be encoded with *D*-dimensional vectors is $\left[0,2^{D-1}-1\right]$ and up to D−1 vectors are required for this encoding. However, among $\left[0,2^{D-1}-1\right]$, the only case that requires D−1 vectors is $2^{D-1}-1$. If we remove this from the range of integers to be encoded, then up to D−2 vectors are sufficient to encode any integer in $\left[0,2^{D-1}-2\right]$. For example, the range $\left[0,126\right]$ can be expressed using only up to six 8-dimensional vectors.

It should be noted that, with the above-mentioned encoding, the number of encoded vectors may vary depending on the integer to be encoded. However, this may raise a security issue as follows: In Section 4, the bid values will be encoded into multiple vectors using $f_{Y}$, and each vector will be encrypted using FHIPE. FHIPE encryption will hide the actual elements in each vector; however, the number of vectors remains the same before and after the encryption. Therefore, if the number of vectors varies according to $V_{Y}$, an attacker may narrow down the possible candidates. Therefore, the number of resulting vectors must be constant and this is the reason encoding of 0 is necessary. Let *N* denote this constant number of vectors. In this work, we use N=D−2 and set the expressible range as $R=[0,2^{D-1}-2]$, where D≥3, so that encoding any integer in *R* may produce *N* vectors. The following is an example of encoding $V_{Y}=96$ with six 8-dimensional vectors:

$$y_{0}=Y\left(64,8\right)=\left\{0,0,0,0,0,0,0,1\right\}$$

$$y_{1}=Y\left(32,8\right)=\left\{0,0,0,0,0,0,1,0\right\}$$

$$y_{2}=y_{3}=y_{4}=y_{5}=Y\left(0,8\right)=\left\{1,0,0,0,0,0,0,0\right\}$$

The process of encoding an integer using *D*-dimensional vectors is shown in Algorithm 2.

**Algorithm 2**$f_{Y}\left(V_{Y},D\right)$: Encoding a value with subroutine *Y*
**Input**:
Value to be encoded $V_{Y}$, vector size *D***Ensure**:
$V_{Y}\in R=\left[0,2^{D-1}-2\right]$ and D≥3**Output**:
Array of vectors $\left[y_{0},y_{1},\ldots ,y_{D-3}\right]$1:N←D−2 {Number of vectors}2:i←D−2 {Highest power of 2}3:n←0 {Current vector}4:**while**n<N**do** {0≤n<N}5: **if** $V_{Y}\geq 2^{i}$ **then**
6:$\;\;y_{n}\leftarrow Y\left(2^{i},D\right)$ {Call Algorithm 1 as a subroutine}7:
  n←n+1
8:
$\;\;V_{Y}\leftarrow V_{Y}-2^{i}$
9: **else if** $V_{Y}=0$ **then**
10:
$\;\;y_{n}\leftarrow Y\left(0,D\right)$
11:
  n←n+1
12: **else**
13:
  i←i−1
14: **end if**
15:
**end while**
16:
**return** $\left[y_{0},y_{1},\ldots ,y_{N-1}\right]$



### 3.3. Subroutine $X_{L}$

The encoding process of $V_{X}$ is denoted as $f_{X}$. This process uses two subroutines, namely $X_{L}$ and $X_{G}$. The output vectors of these two subroutines will be used to identify the “less than” and “greater than” relations, respectively, in the comparison algorithm presented in Section 3.6. Here, we describe the subroutine $X_{L}$ first. The constraint for $X_{L}$ is the same as that for *Y*, i.e., its input is either 0 or a power of 2. The elements of its output vector are either 0 or 1. The decision for the target index position *p* is the same as that of subroutine *Y*, i.e., if the input is $V_{n}=0$, *p* is set to 0. When $V_{n}=2^{i}$, it is computed as p=i+1. However, the difference is in the rules that are used to calculate the elements of the output vector. All the elements with an index less than or equal to *p* are set to 1, and the elements with an index greater than *p* are set to 0. For example, encoding $V_{n}=16$ into a vector with dimension D=8 results in $X_{L}\left(16,8\right)=\left\{1,1,1,1,1,1,0,0\right\}$:


$X_{L}\left(16,8\right)=$
0124816326411111100

Table 2 shows all possible values encoded with $X_{L}$ for D=8.

### 3.4. Subroutine $X_{G}$

The subroutine $X_{G}$ is the same as $X_{L}$, except for the rules that are used to calculate the resulting vector elements., i.e., all the elements with an index greater than or equal to *p* are set to 1 and the elements with an index less than *p* are set to 0. For example, $X_{G}\left(16,8\right)$ results in {0,0,0,0,0,1,1,1}:


$X_{G}\left(16,8\right)=$
0124816326400000111

Table 3 shows all possible values encoded with method $X_{G}$ for D=8.

### 3.5. Encoding Method $f_{X}$

The method $f_{X}$ for encoding a $V_{X}$ value is very similar to $f_{Y}$, which encodes a $V_{Y}$ value. First, $V_{X}$ is partitioned into a sum of powers of 2, and then the terms are sorted from higher-order terms. However, to encode each term, we use both subroutines $X_{L}$ and $X_{G}$ explained in the two previous subsections., i.e., the number of resulting vectors is doubled compared with that of $f_{Y}$. $f_{X}$ supports the same range $R=[0,2^{D-1}-2]$, and the number of vectors is 2N=2(D−2), where *D* is the dimension of the resulting vectors. As in $f_{Y}$, to keep the number of encoded vectors constant, we apply an encoding of 0. For example, the input $V_{X}=11$ can be written as $V_{X}=2^{3}+2^{1}+2^{0}$. Then, each term, (8, 2, and 1) as well as three zeros is encoded independently using $X_{L}$ and $X_{G}$, assuming that the dimension of the resulting vectors is D=8 and the overall number of vectors is 2N=12. As a result, $V_{X}=11$ can be encoded into 12 vectors as follows:

$$f_{X}\left(11,8\right)⟶\left[X_{L}\left(8,8\right),X_{G}\left(8,8\right),X_{L}\left(2,8\right),X_{G}\left(2,8\right),X_{L}\left(1,8\right),X_{G}\left(1,8\right),X_{L}\left(0,8\right),\ldots ,X_{G}\left(0,8\right)\right]$$

This process is detailed in Algorithm 3.

**Algorithm 3**$f_{X}\left(V_{X},D\right)$: Encoding a value with subroutines $X_{G}$ and $X_{L}$

**Input**:
Value to be encoded $V_{X}$, vector size *D***Ensure**:
$V_{X}\in R$ and D≥3**Output**:
Array of vectors $\left[x_{0,L},x_{0,G},x_{1,L},x_{1,G},\ldots ,x_{D-3,L},x_{D-3,G}\right]$1:N←D−2 {Number of vectors}2:i←D−2 {Highest power of 2}3:n←0 {Current vector}4:**while**n<N**do** {0≤n<N}5: **if** $V_{X}\geq 2^{i}$ **then**
6:  $x_{n,L}\leftarrow X_{L}\left(2^{i},D\right)$
7:  $x_{n,R}\leftarrow X_{G}\left(2^{i},D\right)$
8:  n←n+1
9:  $V_{X}\leftarrow V_{X}-2^{i}$
10: **else if** $V_{X}=0$ **then**
11:  $x_{n,L}\leftarrow X_{L}\left(0,D\right)$
12:  $x_{n,R}\leftarrow X_{G}\left(0,D\right)$
13:  n←n+1
14: **else**
15:  i←i−1
16: **end if**
17:
**end while**
18:
**return** $\left[x_{0,L},x_{0,G},x_{1,L},x_{1,G},\ldots ,x_{N-1,L},x_{N-1,G}\right]$



### 3.6. Comparing $V_{X}$ and $V_{Y}$

In this subsection, we explain how to perform a comparison of the encoded $V_{X}$ and $V_{Y}$ using Algorithm 4. Given two arrays $\left[x_{0,L},x_{0,G},\ldots ,x_{N-1,L},x_{N-1,G}\right]$ and $\left[y_{0},y_{1},\ldots ,y_{N-1}\right]$, i.e., encoded $V_{X}$ and $V_{Y}$, the algorithm outputs 1 if $V_{X}\leq V_{Y}$, or 0 otherwise. In lines 4 and 8, the algorithm performs inner product operations and their results are interpreted as follows:

1. $\langle x_{n,L},y_{n}\rangle =0$ means that the *n*th term of $V_{X}$ is less than the *n*th term of $V_{Y}$.
2. $\langle x_{n,G},y_{n}\rangle =0$ means that the *n*th term of $V_{X}$ is greater than the *n*th term of $V_{Y}$.
3. $\langle x_{n,L},y_{n}\rangle =\langle x_{n,G},y_{n}\rangle =1$ means that the *n*th term of $V_{X}$ equals the *n*th term of $V_{Y}$.

The algorithm compares $V_{X}$ and $V_{Y}$ from their higher-order terms. If the first condition above is satisfied in line 5, then it implies that the highest term of $V_{X}$ is less than that of $V_{Y}$, i.e., $V_{X}<V_{Y}$. Thus, the algorithm returns 1. If the second condition above is satisfied in line 9, then it implies that the highest term of $V_{X}$ is greater than that of $V_{Y}$, i.e., $V_{X}>V_{Y}$. Thus, the algorithm returns 0. When both inner products result in 1, i.e., the third condition above is satisfied, the algorithm reaches line 12. To compare the lower-order terms, the algorithm increments the index variable *n* and continues with the next iteration of the ‘while’ loop. The ‘while’ loop repeats until it encounters the first zero in either inner product operation. The first zero determines the output of the algorithm. If the results of all the inner product operations are 1 for n=0,1,…,N−1, it means that $V_{X}=V_{Y}$. In this case, the algorithm reaches line 14 and returns 1.

Now we analyze the time complexity of Algorithm 4. The dominant operations in this algorithm are performed in line 4 and line 8. Line 4 computes the inner product of two *D*-dimensional vectors, and line 8 executes the same operation. If we define an integer multiplication and addition as unit operations, lines 4 and 8 perform O(D) unit operations. As the while loop is iterated up to *N* times, the time complexity of this algorithm is O(DN).

**Algorithm4** Comparison of two values encoded in two input vectors
**Input**:
Encoded $V_{X}$ as $\left[x_{0,L},x_{0,G},x_{1,L},x_{1,G},\ldots ,x_{D-3,L},x_{D-3,G}\right]$, encoded $V_{Y}$ as $\left[y_{0},y_{1},\ldots ,y_{D-3}\right]$,     vector size *D***Ensure**:

D≥3
**Output**:
1 if $V_{X}\leq V_{Y}$; 0 otherwise.1:N←D−2 {Number of vectors}2:
n←0
3:**while**n<N**do** {0≤n<N}4: $r\leftarrow \langle x_{n,L},y_{n}\rangle$
5: **if** r=0 **then**
6:  **return** 17: **end if**
8: $r\leftarrow \langle x_{n,G},y_{n}\rangle$
9: **if** r=0 **then**
10:  **return** 011: **end if**
12: n←n+1
13:
**end while**
14:**return** 1


**Figure 3:**
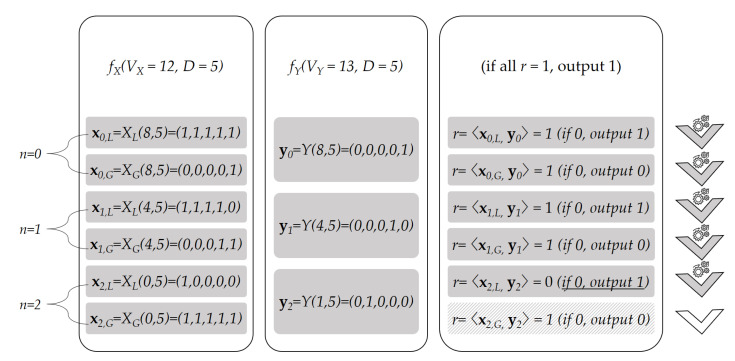
Example with D=5, $V_{X}=12$, $V_{Y}=13$.

Figure 3 illustrates an example of Algorithm 4 with $V_{X}=12$ and $V_{Y}=13$. As the vector dimension is D=5, we need six vectors for $V_{X}$ and three vectors for $V_{Y}$. The figure shows which vectors are used to compute *r* for each iteration n=0,1,2. As $V_{X}$ and $V_{Y}$ are decomposed as $V_{X}=8+4+0$ and $V_{Y}=8+4+1$, they tie for n=0 and n=1. The first r=0 appears in line 5 when n=2. The algorithm outputs 1, indicating that $V_{X}\leq V_{Y}$.

## 4. Proposed Energy Trading System

### 4.1. System Components

**Figure 4:**
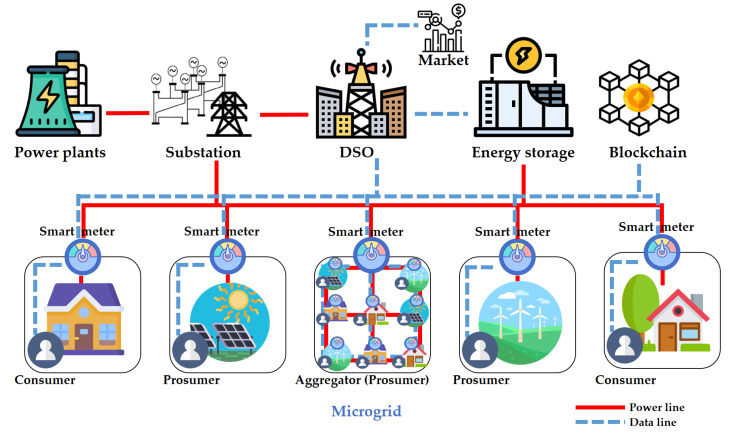
Proposed energy trading system model.

To verify the feasibility of the new encoding method, we implemented a prototype energy trading system. Our system is composed of a DSO, energy storage, blockchain, and prosumers with their smart meters, and these participants are interconnected as in Figure 4. The system model and security policies of the proposed system are similar to those in [13]. Following the model in [12,53], we consider a smart meter as a sealed tamper-proof device. Therefore, even a prosumer who owns a smart meter cannot manipulate the secret key installed in the device. Smart meters also play the role of a network node that connects prosumers with the DSO and the blockchain. The smart meters have a software module that generates bid requests to buy and sell electricity. The bidding price is determined using a pre-trained machine learning model that performs regression based on the previous electricity prices. We omit the details for this regression model, as it is out of the scope of this paper. As most of the current electricity markets adopt the day-ahead pricing method, we apply a bidding policy where smart meters generate bid requests for each hour of the next day, depending on today’s feedback. However, we remark that the proposed encoding and secure comparison methods are not restricted to this policy as they are orthogonal to bidding policies. The software module in each smart meter also encrypts the bid requests using the left and right encryption algorithms of the FHIPE scheme explained in Section 2.2.

The energy storage and smart meters are interconnected through a local distribution network so that bidirectional energy flow is possible. The energy storage is assumed to be a trusted party and honestly performs the functions requested by the DSO. A DSO handles the power transmission from power plants to local prosumers through a transmission line and a substation. It also handles the bidirectional power distribution between the smart meters and the energy storage. However, in this paper, we do not deal with the details of the physical power lines.

The blockchain receives the encrypted bid values from the smart meters. It maintains an encrypted priority queues and performs secure matching of the bid values by repeating the secure comparison on encrypted bid vectors, i.e., Algorithm 4 is performed repeatedly on encrypted vectors for $V_{X}$ and $V_{Y}$. The details of this process are explained in the next subsection.

When a match occurs, the actual transmission of powers and the settlement of the balance of each prosumer are handled by the DSO. Prosumers have a registered account in the DSO. Consequently, the DSO knows the identity of the users for accounting and billing purposes. However, it cannot forge the energy transactions because all the matching transactions are performed on the blockchain. In addition, no party other than the DSO can obtain any information about the identity or bid prices of the prosumers as these are protected using a one-time identifier *OID* and FE, respectively. This is achieved by implementing a smart contract on the blockchain that uses FE.

### 4.2. Matching Algorithm

In this subsection, we describe how blockchain uses the proposed algorithm for matching a seller and a buyer using their encoded prices. Then, we explain how to achieve a privacy-preserving matching algorithm by applying FE to the encoded prices. Let $pp_{U}$ be the price for trading of a user U∈S∪B, where **S** and **B** are the set of sellers and the set of buyers, respectively. The matching algorithm is implemented as in traditional auctions or stock markets, such that the buyer with the highest bid price, denoted as $B_{max}$, is matched with the seller who has the lowest price, denoted as $S_{min}$. Therefore, matching is impossible if $pp_{S_{min}}>pp_{B_{max}}$. When $S_{min}$ and $B_{max}$ are matched, the power amount for the trade is determined as $min(pa_{S_{min}},pa_{B_{max}})$, where $pa_{U}$ is the power amount stated by user *U*. We use two array-based heap data structures, a min-heap $H_{S}$ and a max-heap $H_{B}$ for the sellers and buyers, respectively, to locate $S_{min}$ and $B_{max}$ easily by using the bid values as the primary keys. Consequently, the roots of $H_{S}$ and $H_{B}$ hold $S_{min}$ and $B_{max}$, respectively. According to the property of the heaps, the time complexity for inserting and deleting a bid is $O(log_{2}|S|)$ for the sellers and $O(log_{2}|B|)$ for the buyers. To guarantee the privacy of the sellers and buyers, we will encrypt the nodes of these heaps using FHIPE. The details of the node encryption and comparison of encrypted bids are explained in the next paragraph.

**Figure 5:**
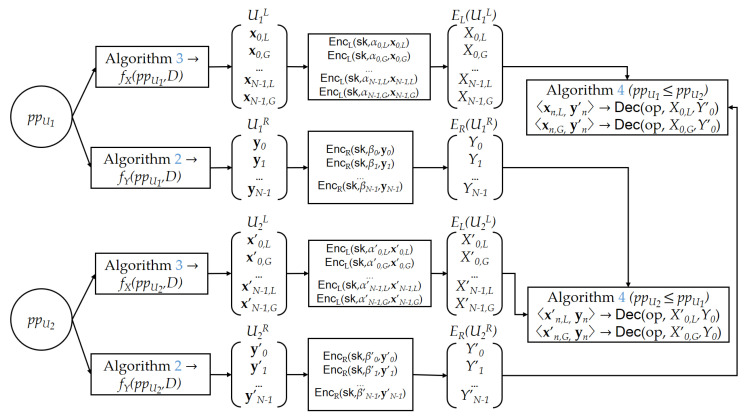
Comparison of encrypted bids.

To maintain and update the heaps, and to perform peer matching, we use the proposed encoding and comparison algorithms. Figure 5 shows the overall procedure for bid encoding, encryption, and comparison. The vector size *D* is decided as a global parameter according to the value range appropriate for the needs of an application. The bid $pp_{U}$ of user *U* is encoded into two sets of *D*-dimensional vectors, $U^{L}$ and $U^{R}$, using Algorithms 2 and 3, respectively, i.e., $U^{L}$ contains 2N*D*-dimensional vectors and $U^{R}$ contains *ND*-dimensional vectors. When the two values, $pp_{U_{1}}$ and $pp_{U_{2}}$, are encoded as $\left(U_{1}^{L},U_{1}^{R}\right)$ and $\left(U_{2}^{L},U_{2}^{R}\right)$, respectively, the comparison of $pp_{U_{1}}$ and $pp_{U_{2}}$ can be performed using Algorithm 4, taking as input $U_{1}^{L}$ and $U_{2}^{R}$, i.e., we can set $\left[x_{0,L},x_{0,G},x_{1,L},x_{1,G},\ldots ,x_{N-1,L},x_{N-1,G}\right]\leftarrow U_{1}^{L}$ and $\left[y_{0},y_{1},\ldots ,y_{N-1}\right]\leftarrow U_{2}^{R}$, and perform the algorithm. If the output is 1, it implies that $pp_{U_{1}}\leq pp_{U_{2}}$. ( Alternatively, we may use $U_{2}^{L}$ and $U_{1}^{R}$ for comparison.) However, the actual comparison of two bids is not done using $U_{1}^{L}$ and $U_{2}^{R}$. To preserve the privacy of the users, we perform the comparison on a ciphertext domain. To make this possible, the users’ smart meters provide encrypted vectors of the encoded bid values. Before submitting a bid, the smart meter encrypts the encoded bid value using FHIPE [44]. This encryption is performed element-wise, i.e., for $U^{L}=\left[x_{0,L},x_{0,G},x_{1,L},x_{1,G},\ldots ,x_{N-1,L},x_{N-1,G}\right]$ that has been encoded by Algorithm 3, the user *U*’s smart meter computes the list of left encryptions $E_{L}\left(U^{L}\right)$ = [${\mathrm{Enc}}_{L}(\mathrm{sk},\alpha_{0,L}$,$x_{0,L}$), ${\mathrm{Enc}}_{L}(\mathrm{sk},\alpha_{0,G}$,$x_{0,G}$), ${\mathrm{Enc}}_{L}(\mathrm{sk},\alpha_{1,L}$,$x_{1,L}$), ${\mathrm{Enc}}_{L}(\mathrm{sk},\alpha_{1,G}$,$x_{1,G}$), …, ${\mathrm{Enc}}_{L}(\mathrm{sk},\alpha_{N-1,L}$,$x_{N-1,L}$), ${\mathrm{Enc}}_{L}(\mathrm{sk},\alpha_{N-1,G}$,$x_{N-1,G}$)] using random elements $\alpha_{0,L},\alpha_{0,G},\ldots ,$$\alpha_{N-1,L},\alpha_{N-1,G}\in Z_{q}$. This process requires 2N applications of ${\mathrm{Enc}}_{L}$. As each ${\mathrm{Enc}}_{L}$ performs approximately *D* point multiplications on the elliptic curve group $G_{1}$, 2DN point multiplications are required in total. The list of right encryptions $E_{R}\left(U^{R}\right)$ can be computed similarly by applying the ${\mathrm{Enc}}_{R}$ function independently to each element in $U^{R}=\left[y_{0},y_{1},\ldots ,y_{N-1}\right]$ that has been encoded by Algorithm 2. This process requires DN point multiplications on the elliptic curve group $G_{2}$.

Then, the user *U* submits its encrypted bid as a selling or buying bid. Selling bids are inserted into the min-heap and buying bids into the max-heap as a pair $\left(E_{L}\left(U^{L}\right),E_{R}\left(U^{R}\right)\right)$. We implement a heap as an array, where each element holds the encrypted value of a bid $\left(E_{L}\left(U^{L}\right),E_{R}\left(U^{R}\right)\right)$ and the auxiliary data. Let $EH_{S}$ and $EH_{B}$ be the two heaps that hold encrypted bids for sellers and buyers, respectively. The comparison of encrypted bids depicted in Figure 5 is used for two purposes. First, it is used to compare the nodes inside an encrypted heap (either $EH_{S}$ or $EH_{B}$) to maintain the heap property when inserting or deleting an encrypted bid. Second, it is used to find a match between the two top elements in $EH_{S}$ and $EH_{B}$. The decryption operation of FHIPE is used for this comparison. As the result of the decryption operation $\mathrm{Dec}(\mathrm{op},{\mathrm{Enc}}_{L}\left(x\right),{\mathrm{Enc}}_{R}\left(y\right))$ is equivalent to 〈x,y〉, we can perform the comparison in the ciphertext domain by slightly changing Algorithm 4. For this purpose, the input to the algorithm is replaced by two sets of encrypted vectors of the bid value, i.e., $E_{L}\left(U^{L}\right)$ and $E_{R}\left(U^{R}\right)$, and additional public parameters op are passed. Then, the inner product operation is replaced by the decryption operation of FHIPE. Let $\left(E_{L}\left(U_{1}^{L}\right),E_{R}\left(U_{1}^{R}\right)\right)$ and $\left(E_{L}\left(U_{2}^{L}\right),E_{R}\left(U_{2}^{R}\right)\right)$ be the encrypted bid values from users $U_{1}$ and $U_{2}$, respectively. If ($E_{L}\left(U_{1}^{L}\right),E_{R}\left(U_{2}^{R}\right)$) are given as arguments for the updated comparison algorithm, and the output is 1, it means that $pp_{U_{1}}\leq pp_{U_{2}}$, otherwise, $pp_{U_{1}}>pp_{U_{2}}$. We denote the updated comparison algorithm as COMP($E_{L}\left(U_{1}^{L}\right),E_{R}\left(U_{2}^{R}\right)$). We can see that the number of pairing operations for COMP is 2DN in the worst case if we apply a reasoning similar to the analysis of Algorithm 4.

We adopt Algorithm 5 (INSERT procedure) from [13], which has been used to insert a new bid element into $EH_{S}$. The INSERT procedure calls COMP as a subroutine to sort the elements and restore the heap. Insertion of a new bid element into $EH_{B}$ is performed by changing line 6 of Algorithm 5 as $\mathrm{COMP}(EH_{B}\left[\left\lfloor idx/2\right\rfloor \right].E_{L},EH_{B}\left[idx\right].E_{R})=1$. Heaps are implemented as a typical complete binary tree such that the root node is at EH[1]. The parent node of EH[i] is at EH[⌊i/2⌋], and its left and right children are at EH[2i] and EH[2i+1], respectively. We also adopt the REMOVEMIN($EH_{S}$) procedure from [13] to remove the minimum, i.e., the root element of the heap $EH_{S}$. Finally, we also adopt Algorithm 6 (MATCHING procedure) from [13] to find a possible match.

**Algorithm 5** INSERT procedure for min-heap $EH_{S}$ [13]
**Input**:
$E_{L}\left(U^{L}\right),E_{R}\left(U^{R}\right)$, auxiliary data**Output**:
None1:$idx\leftarrow (\mathrm{size}\;\mathrm{of}\;\left(EH_{S}\right))+1$ {Insert the new item as the last leaf node}2:
$EH_{S}\left[idx\right].E_{L}\leftarrow E_{L}\left(U^{L}\right)$
3:
$EH_{S}\left[idx\right].E_{R}\leftarrow E_{R}\left(U^{R}\right)$
4:
$EH_{S}\left[idx\right].aux\leftarrow \mathrm{auxiliary}\;\mathrm{data}$
5:**while**idx>1**do** {Perform *upheap* to restore the heap order}6: **if** $\mathrm{COMP}(EH_{S}\left[idx\right].E_{L},EH_{S}\left[\left\lfloor idx/2\right\rfloor \right].E_{R})=1$ **then**
7:
$\;\;\mathrm{swap}\;EH_{S}\left[idx\right]\;\mathrm{and}\;EH_{S}\left[\left\lfloor idx/2\right\rfloor \right]$
8:
  idx←⌊idx/2⌋
9: **else**
10:  **break**
11: **end if**
12:
**end while**



**Algorithm 6** MATCHING procedure to check for possible match [13]
**Input**:
None**Output**:
data or *false*1:
**if** $\mathrm{COMP}(EH_{S}\left[1\right].E_{L},EH_{B}\left[1\right].E_{R})=1$ **then**
2:
$\;S_{min}\leftarrow \mathrm{REMOVEMIN}\left(EH_{S}\right)$
3:
$\;B_{max}\leftarrow \mathrm{REMOVEMIN}\left(EH_{B}\right)$
4: **return** $S_{min}.E_{L},S_{min}.E_{R},S_{min}.aux\;\mathrm{and}\;B_{max}.E_{L},B_{max}.E_{R},B_{max}.aux$
5:
**else**
6: **return** false
7:
**end if**



### 4.3. Proposed Energy Trading Protocol

In this section, we explain our new protocol for performing privacy-preserving energy trading. Basically, the proposed protocol is based on the protocol presented in [13]. However, we introduce a finite state machine managed in the blockchain to provide a rebidding functionality, which has not been provided in [13].

#### 4.3.1. Setup Stage

In our protocol, the DSO generates a secret key sk and public parameters op using the Setup algorithm discussed in Section 2.2. In addition, the DSO deploys a smart contract on the blockchain with the public parameters op. Any party can check the validity of the smart contract. We assume that all prosumers have an account registered with the DSO and a corresponding permanent user identifier *UID*. Their smart meters store the pre-shared secret key sk generated by the DSO and the address of the smart contract. We also assume that all parties agree on a constant *D* (the dimension of the vectors) that is determined by the DSO and that defines the valid range *R* of price and the number of vectors *N* used for encoding.

#### 4.3.2. Finite State Machine in the Blockchain

**Figure 6:**
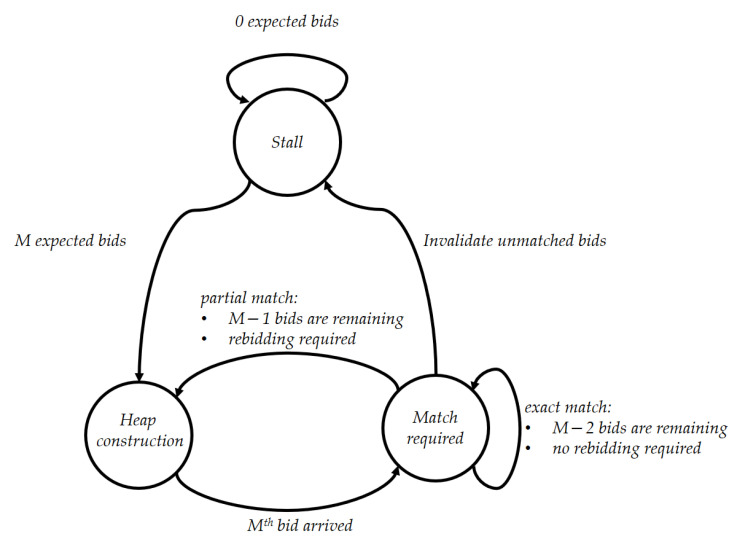
Finite State Machine.

**Figure 7:**
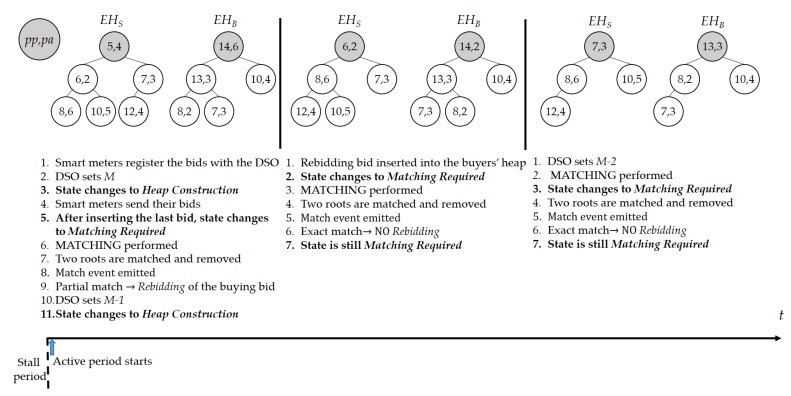
Heap management.

We implement our smart contract as a finite state machine with three states: *Stall, Heap Construction*, and *Match Required*, as shown in Figure 6. State changes in the smart contract are triggered either by specific conditions or by the DSO. Basically, there are two periods for the application: active period and stall period. At the beginning of the active period, smart meters register their (*UID, OID*) relation to the DSO, where OID is a one-time ephemeral identifier generated and used for each session. After the smart meters complete sending these pairs, the DSO counts the number of pairs and sets this number as the number of bids that it expects to receive. Let *M* be this number. The state of the smart contract is set to *Heap Construction*, indicating that it is constructing heaps. Smart meters can send their encrypted bids to the blockchain only when the smart contract is in the *Heap Construction* state. Every time a new encrypted bid arrives, the smart contract updates either $EH_{S}$ and $EH_{B}$, depending on whether the bid is a selling or a buying bid. When the sum of the numbers of bids in $EH_{S}$ and $EH_{B}$ becomes equal to *M*, the state of the smart contract is changed to *Match Required*, indicating that it has already gathered all expected bids. Then, the MATCHING procedure is performed and the root nodes of $EH_{S}$ and $EH_{B}$ are securely compared. The MATCHING procedure can be performed only when the smart contract state is *Match Required*. When the MATCHING procedure is successful, i.e., two bids are matched, a *Match* event is emitted. If the declared power amounts $pa_{S}$ and $pa_{B}$ of a matched seller and buyer are exactly the same, the two matching parties will be able to trade the exact amount of electricity. This exact match reduces the total number of nodes in $EH_{S}$ and $EH_{B}$ by two, and *M* is decreased by two. As the new root nodes of the updated heaps should be compared by calling the MATCHING procedure once again, the state of the blockchain remains as *Match Required*. However, if $pa_{S}\neq pa_{B}$, only the amount $\min(pa_{S},pa_{B})$ can be traded. Therefore, an additional matching is required for the remaining amount. We handle this by letting the owner of the remaining bid send a new encrypted bid with the remaining amount. We call this process *rebidding*. To prevent a possible misconduct of the user, e.g., changing the amount of the remaining bid when performing rebidding, the DSO will verify the validity of the bids, as explained in the next subsection. When rebidding is required, *M* is decreased by only one, instead of two. As the two root nodes were removed from the heaps for the initial matching, and *M* decreased by only one, the blockchain will be waiting for an additional bid and the state of the blockchain reverts to *Heap Construction*. When the expected additional bid arrives, the blockchain will transition to the *Match Required* state and additional matching will be tried. When no more MATCHING is successful, the heaps are not updated any more. At the end of the active period, all the unmatched bids in the heaps are invalidated. In addition, to inform the smart meters of the unmatched bids, the blockchain emits an *Invalidation* event, which includes the *OID*s of the invalidated bids. Now the stall period starts. Figure 7 shows an example of state changes and updates of two encrypted heaps when bidding, matching, and rebidding operations are performed. Each node in the heaps represents a bid from a user and it includes the pair (pp,pa), i.e., the power price and amount that the corresponding user has declared for trading. Even though the example shows the internal values of each node, the actual values are protected by FHIPE.

#### 4.3.3. Bidding and Matching Operations

**Figure 8:**
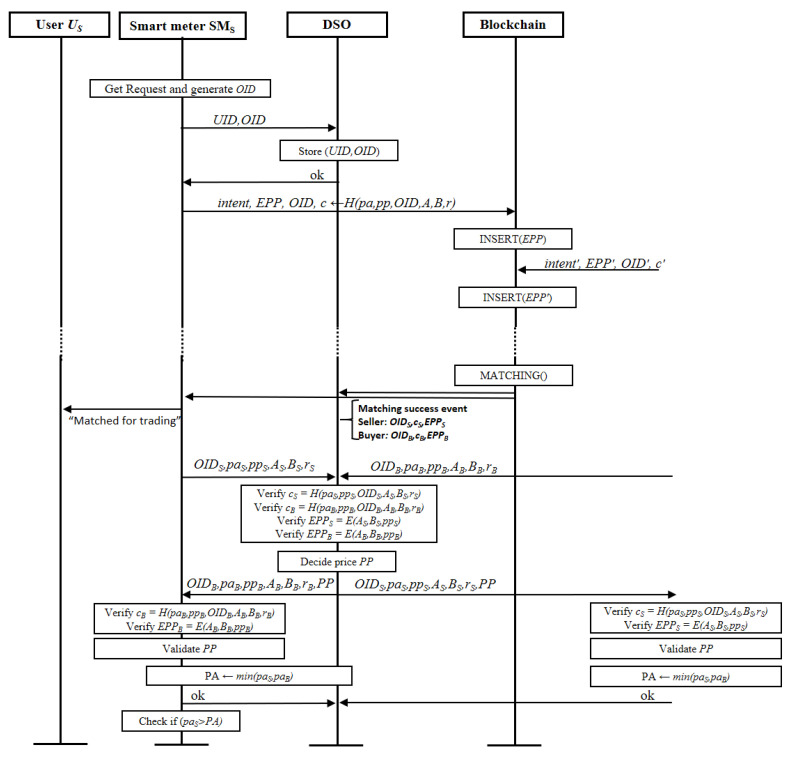
Bidding and matching operations.

Figure 8 illustrates the bidding and matching operations of our protocol. First, the smart meter decides an appropriate bid value to request using its internal machine learning model. The bid request consists of the *intent* to “buy” or “sell,” power amount pa∈Z that needs to be sold or bought, and power price pp∈R for each unit of energy power, where *R* is the range of valid bid values. A smart meter generates a random *OID* for a new session, which is used to hide the real identity *UID* of the prosumer. The smart meter sends the generated *OID* to the DSO to register the relation between *UID* and *OID*. Then, the smart meter encodes *pp* into multiple vectors and encrypts them as explained in Section 4.2. Encryption of *pp* creates two sets of ciphertexts, one for ${\mathrm{Enc}}_{L}$ and the other for ${\mathrm{Enc}}_{R}$. We denote them as *EPP* collectively. In addition, the sets of α and β values used in the encryption are denoted as *A* and *B*, respectively. In addition, the smart meter calculates *c*, which is the hash value of *pa, pp, OID, A, B*, and a random *r*, as a commitment.

The smart meter sends *intent, EPP, OID*, and *c* to the smart contract on the blockchain that has been deployed by the DSO and verified by all blockchain nodes during the setup phase. The smart contract performs the INSERT procedure explained in Algorithm 5 to insert the bid element into one of the heaps according to its *intent*. At the same time, many other bids are sent from multiple smart meters. The blockchain accepts *M* bids, which is the expected bid count. After inserting the last bid, the smart contract performs a matching operation. If there is a match, a blockchain event will be emitted with the *EPP* and the auxiliary data of $S_{min}$ and $B_{max}$. The smart meter implements an event listener and is aware of every *Match* event. The two smart meters corresponding to the two matched bids learn that they were selected for trading, by checking the OIDs in the *Match* event. They open to the DSO the data *OID, pa, pp, A, B*, and *r* that have been used for the bidding, and then the DSO checks the validity of the data by verifying that *EPP* and commitment *c* are correctly reproduced from these data. After validating the data from both the seller and the buyer, by calculating the hash value H(pa,pp,OID,A,B,r) and the EPP using the provided α and β values, the DSO computes the price for the trade as $PP=(pp_{S}+pp_{B})/2$. Then, the DSO forwards the seller’s data to the buyer, along with *PP*, and the buyer’s data to the seller. Both parties validate each other’s data and decided price *PP*. Then, the two smart meters and the DSO calculate the trading amount of power as $PA\leftarrow min(pa_{S},pa_{B})$. If all verification and other processes are completed successfully, the smart meters send an “ok” message to the DSO. Next, both smart meters check whether the decided power amount is less than its desired amount, i.e., pa>PA, and if so, a new bidding and matching process starts from the beginning with the remaining amount pa−PA, which we already defined as rebidding.

#### 4.3.4. Actual Trading

**Figure 9:**
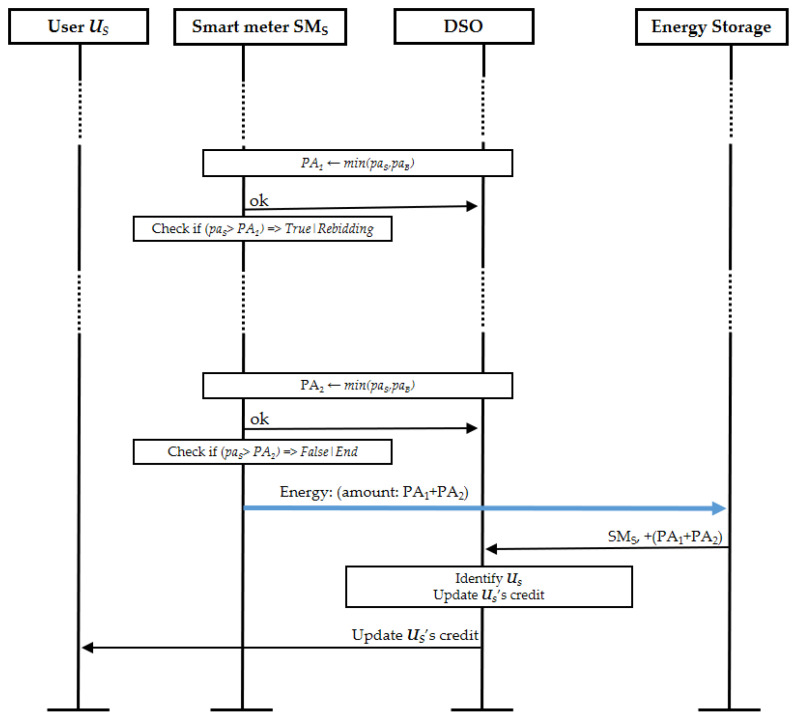
Trading operation.

Finally, we briefly explain the trading operation in the proposed protocol. Figure 9 illustrates an example trading operation for a selling user $U_{S}$ that sold its energy in two parts, i.e., after the initial bidding and the first match, user $U_{S}$ had the remaining amount of energy and performed a rebidding. Then, when the second bid of user $U_{S}$ was matched, the full remaining amount of energy was successfully sold. When the active period ends, the smart meter $SM_{S}$ of user $U_{S}$ feeds the energy amount of ${\mathrm{PA}}_{1}+{\mathrm{PA}}_{2}$ to the energy storage. Consequently, buyers consume the amount of energy they bought from the energy storage after the active period ends. The amount of energy fed by the seller or consumed by the buyer is reported to the DSO by the energy storage. Then, the DSO updates the prosumers’ credit and debit for billing purposes and notifies the users about their balances.

## 5. Performance Analysis

In this section, we verify the efficiency of the proposed encoding algorithm and the feasibility of the proposed system by implementing the system prototype. The prototype is composed of a DSO, five smart meters, and a private blockchain network with ten nodes. We implemented the DSO on a desktop PC with an Intel Core i7-7700 CPU @ 3.60 GHz and 16 GB of main memory. For the Ethereum private network with 10 nodes, we used Amazon Web Services EC2 t2.medium type servers. We used five Raspberry Pi 2 devices with a 900-MHz quad-core ARM Cortex-A7 CPU and 1 GB of RAM to simulate smart meters. We used the Python programming language to implement the software for the DSO and the smart meters. We also used the Solidity language to implement the smart contracts [54]. The FHIPE modules were adopted from [55] for the DSO and from [13] for the smart meter, where both of them implement an optimal Ate pairing [56] on a pairing-friendly Barreto-Naehrig curve [57]. Both implementations have applied optimizations for the pairing-based cryptography proposed in [58,59]. The only difference between the two is that the FHIPE module for DSO additionally adopted parallel processing techniques. Go implementation of the Ethereum protocol, Geth(go-ethereum), was used as an Ethereum client for the blockchain.

### 5.1. Performance Analysis of the Proposed Algorithm

**Figure 10:**
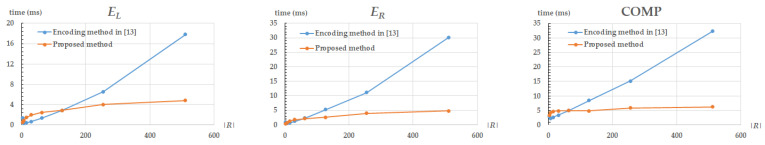
Comparison of the computation times of the $E_{L}$, $E_{R}$ and COMP on the DSO.

**Figure 11:**
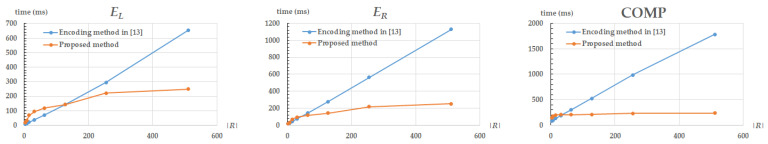
Comparison of the computation times of the $E_{L}$, $E_{R}$ and COMP on the smart meter.

In this subsection, we show the performance evaluation results of the proposed encoding algorithm compared with the encoding method used in [13] in the context of FHIPE using the DSO and a smart meter. Figure 10 and Figure 11 compare the computation times of the $E_{L}$, $E_{R}$ and COMP on the DSO and the smart meter, respectively. The $E_{L}$, $E_{R}$ and COMP repeatedly use left encryption (${\mathrm{Enc}}_{L}$), right encryption (${\mathrm{Enc}}_{R}$), and decryption (Dec) operations of the FHIPE module. We do not provide the data for the Setup operation of FHIPE, as it is executed only once, and can be performed offline. We included the figures for COMP for reference, although neither the DSO nor the smart meter performs it. (Only the blockchain performs COMP function.) The $E_{L}$ and $E_{R}$ are computed by the smart meters to encode the power price pp and to verify the matched bid information that is passed by the DSO. In addition, the DSO also computes them for verification of the encrypted bids.

We tried various sets for *R*, where *R* is the range of integers that can be encoded. To be precise, we performed experiments with |R|=7,15,31,63,127,255, and 511. For each *R*, we measured the computation time for 1000 runs and calculated the 10% trimmed mean to evaluate the performance of the two encoding methods. We observed that the previous encoding method proposed in [13] is faster for small ranges; however, for a more practical setup with a greater *R*, all operations were completed faster with the proposed encoding, and the performance gain increased for greater ranges. (The break-even point is |R|=127 for $E_{L}$ and |R|=63 for $E_{R}$ and COMP.) For example, for |R|=511, the computation times of the $E_{L}$, $E_{R}$, and COMP operations using the proposed method were shorter than those in [13] by 3.70, 6.25 and 5.22 times, respectively on the DSO. This is because a unary encoding was used in [13], i.e., the number of elements in a vector was O(|R|) in [13]. Please note that the computation times of $E_{L}$ and $E_{R}$ are almost proportional to the number of vector elements given as input because each vector element requires a point multiplication on an elliptic curve group. Therefore, $E_{L}$ and $E_{R}$ in [13] requires O(|R|) point multiplications. Similarly, the number of paring operations for COMP in [13] is O(|R|). On the contrary, according to the analysis in Section 4.2, the computation of $E_{L}$ and $E_{R}$ using the proposed method requires 2DN and DN point multiplications, respectively, which correspond to $O({\left(\log|R|\right)}^{2})$ as N=D−2 and D=O(log|R|). In the worst case, COMP in the proposed method also requires 2DN pairing operations as analyzed in Section 4.2. This is also $O({\left(\log|R|\right)}^{2})$, which is significantly smaller than O(|R|).

A concrete example is provided below. To represent |R|=511 different integers (R=[0,510]), the encoding method of [13] uses two vectors with 511 elements, one for $E_{L}$ and the other for $E_{R}$. Therefore, the $E_{L}$ and $E_{R}$ required 511 point multiplications each and COMP required 511 pairing operations. Conversely, in our proposed encoding method, $V_{X}$ is encoded twice, producing two vector lists with DN elements, and $V_{Y}$ is encoded into one list with DN elements. For |R|=511, we can use D=10, thus N=8. Therefore, the $E_{L}$ and $E_{R}$ requires 2×10×8=160 and 10×8=80 point multiplications, respectively. The maximum number of pairings for the COMP is 2×10×8=160, but the measured values are less than this in most cases, as the ‘while’ loop of Algorithm 4 may exit before iterating *N* times.

### 5.2. Performance Analysis of the Proposed System

In this subsection, we verify the feasibility and show the practicality of the proposed system. For the experimental setting, we set both the active and the stall period to 2 min, i.e., tradings are repeated in a 4-min cycle. This setup is a very harsh condition compared to real-world systems. In a real-world scenario, the tradings are performed on an hourly basis, i.e., the prosumers are able to take part in energy trading every hour with, for example, a 10-min active period and 50-min stall period. According to the rate plans by the Korea Electric Power Corporation (KEPCO) [60], the electricity charge was up to 275.6 KRW/kWh. However, FHIPE operations [44] and the proposed encoding algorithm are defined over integers. To avoid losing significant digits, in our case one decimal point, we quantized the input, i.e., power price, as pp×10. The dimension of the resulting vectors was set to D=13, where the range of price is R∈[0,4094] to cover the integers up to 2756, which is the maximum electricity price range after applying quantization. With this setup, the protocols between the DSO, five smart meters, and a private blockchain network with ten nodes finished the trading process within 2 min, even in the worst case. By the worst case, we mean that every prosumer wants to participate in trading at the same time and the maximum number of matches and rebidding occurs. As shown in Figure 7, one execution of the MATCHING procedure (Algorithm 6) reduces the number of heap nodes by at least one. Therefore, there can be up to 4 matches, and 3 rebidding processes are performed in the worst-case scenario. For example, there is one seller with pa=10 and pp=100, and four buyers with pa=2 and pp≥100. In this case, the seller is going to bid 4 times, i.e., the initial bid and 3 additional bids for rebidding. In general, there will be up to l−1 matches, where *l* is the number of prosumers.

To quantify the work that the blockchain does and to estimate code complexity, gas consumption is used as the most reliable evaluation metric. Table 4 shows the gas consumption of two dominant operations of our protocol, namely the creation of a heap node and the COMP operation, and compares it with that of the previous work [13]. We measured the average gas cost for these operations for 100 iterations with a random power price pp and power amount pa. The heap node creation is performed using Algorithm 5, and storing a bid that contains ciphertexts for the encrypted bid value and the auxiliary data requires a non-negligible amount of memory. In the case of the COMP operation, we used precompiled contracts of Geth. We need to point out that we used the Istanbul fork [61] in our Ethereum private network because this upgrade introduces significant gas cost reduction for the pairing check operation by optimizing the underlying elliptic curve operations. For example, the gas cost for the pairing check operation using the precompiled contract at address 0×08 involving *D*-dimensional vectors is 34,000 ×D + 45,000 [62]. Now we compare the efficiency of our proposed encoding operations with previous work [13]. In the previous work, the gas cost for heap node creation was linear in the range of elements, roughly 133,424 ×|R| + 292,000, which is not significant for small ranges. However, for greater |R|, an immense quantity of gas may be required. For example, |R|=4095 would require approximately 546 million worth of gas, whereas our solution is approximately 20.63 times less costly. The implementation of the COMP operation may require a different amount of gas depending on the input and the measurements in Table 4 are for random values. We remark that the previous work did not apply the updated precompiled contracts of the Istanbul fork, i.e., its gas cost for the pairing check operation involving *D*-dimensional vectors is 80,000 ×D + 100,000 [63], which is roughly 2.35 times greater than that in [62]. For a fair comparison, we included a normalized ratio as the last column. We can see that even with this normalization considered, the proposed method uses significantly fewer resources compared to [13]. Besides the performance gain, the proposed method also provides scalability because the gas cost does not increase linearly as |R| increases.

## 6. Discussion

According to the analysis in the previous section, the new encoding method and algorithm significantly reduce the complexity of FE operations. Consequently, they reduce the gas cost required to perform trading operations on the blockchain, which proves that the proposed solution is more practical and scalable compared to the previous work [13]. However, there are still some limitations and challenges in the proposed method. The block gas limit of the Ethereum main network is approximately 12.5 million at the time of writing. Although the proposed method dramatically reduces the gas cost, it is still too high for deployment on the Ethereum main network. Please note that we used dual vectors $x_{n,L}$ and $x_{n,R}$ in lines 6 and 7 of Algorithm 3 for a single two’s power term because the comparison should be applied following two binary decisions. Therefore, Algorithm 4 conducts two inner product computations (in line 4 and line 8) to determine one result from three possibilities: <,=, and >. We applied this design as the underlying precompiled contract in Ethereum for the pairing product operation used in the FHIPE decryption only returns a binary output, i.e., either 0 or 1. Therefore, if we can improve the precompiled contract for it to produce, e.g., a ternary output, we may then reduce the required computation by half using the ternary encoding algorithm [52]. Furthermore, the introduction of the rebidding feature limits the number of matched bids per block to only one. After every price matching of two bids, the matched amount is checked outside of the blockchain and it is decided whether rebidding is required according to the remaining amount of energy. This limits the number of matches that can be performed in a block. Therefore, it would be better if we could handle multiple matches in one block by allowing the decision made inside the blockchain. In addition, scalability could be evaluated with more participants. In this paper, we have considered five smart meters for our testbed. It would be interesting how the performance varies when more smart meters are involved. Finally, it would be possible to consider a relaxed security requirement. In the current design, both the bid price and the identity of each trading participant are protected. However, in a certain scenario, electricity prices can be public in an open market, and the only security requirement would be the protection of identity. In this case, bid values need not be encrypted, and the combination of OID and cryptographic hash functions are sufficient, which dramatically reduces gas cost. The decision on the exact security requirement should be performed according to the structure of the energy market, relevant regulations and privacy policy. We leave these issues for future research.

## 7. Conclusions

The combination of renewable energy systems and blockchain-based P2P energy trading may provide a more stable energy market with cheaper energy sources. However, for this to be acceptable to the public, the security and privacy of all parties must be guaranteed. Although a blockchain inherently provides data integrity and is able to eliminate the central trusted party, the privacy aspect requires further investigation. In this study, we constructed a new vector encoding algorithm to perform an efficient and secure integer comparison using multiple inner products for inner product encryption. We applied the new encoding method to design a privacy-preserving P2P energy trading system. We also improved the previous protocol by considering rebidding for the remaining amount of energy. To verify the feasibility of the proposed system, we implemented a prototype composed of a DSO, smart meters, and a private Ethereum blockchain, and conducted a field test with various parameters. According to our analysis, the new encoding algorithm significantly improves the performance of trading operations. However, as discussed in the previous section, the proposed system can be improved in various aspects, e.g., the further reduction of gas cost, handling multiple matches in a single block, scalability evaluation with more participants, and consideration of various security policies. We leave these issues for future research.

## Figures and Tables

**Table 1:** All possible values encoded with method *Y*.

D = 8	0	1	2	4	8	16	32	64
Y(0,8)	1	0	0	0	0	0	0	0
Y(1,8)	0	1	0	0	0	0	0	0
Y(2,8)	0	0	1	0	0	0	0	0
Y(4,8)	0	0	0	1	0	0	0	0
Y(8,8)	0	0	0	0	1	0	0	0
Y(16,8)	0	0	0	0	0	1	0	0
Y(32,8)	0	0	0	0	0	0	1	0
Y(64,8)	0	0	0	0	0	0	0	1

**Table 2:** All possible values encoded with method $X_{L}$.

D = 8	0	1	2	4	8	16	32	64
$X_{L}\left(0,8\right)$	1	0	0	0	0	0	0	0
$X_{L}\left(1,8\right)$	1	1	0	0	0	0	0	0
$X_{L}\left(2,8\right)$	1	1	1	0	0	0	0	0
$X_{L}\left(4,8\right)$	1	1	1	1	0	0	0	0
$X_{L}\left(8,8\right)$	1	1	1	1	1	0	0	0
$X_{L}\left(16,8\right)$	1	1	1	1	1	1	0	0
$X_{L}\left(32,8\right)$	1	1	1	1	1	1	1	0
$X_{L}\left(64,8\right)$	1	1	1	1	1	1	1	1

**Table 3:** All possible values encoded with method $X_{G}$.

D = 8	0	1	2	4	8	16	32	64
$X_{G}\left(0,8\right)$	1	1	1	1	1	1	1	1
$X_{G}\left(1,8\right)$	0	1	1	1	1	1	1	1
$X_{G}\left(2,8\right)$	0	0	1	1	1	1	1	1
$X_{G}\left(4,8\right)$	0	0	0	1	1	1	1	1
$X_{G}\left(8,8\right)$	0	0	0	0	1	1	1	1
$X_{G}\left(16,8\right)$	0	0	0	0	0	1	1	1
$X_{G}\left(32,8\right)$	0	0	0	0	0	0	1	1
$X_{G}\left(64,8\right)$	0	0	0	0	0	0	0	1

**Table 4:** Gas cost comparison for the heap node creation and the COMP operation.

	**Consumed** **Gas**	**(A) Heap Node** **Creation in [13]**	**(B) Heap Node** **Creation** **(proposed)**	**(C) Ratio** **(A)/(B)**	**(D)** **COMP** **in [13]**	**(E)** **COMP** **(proposed)**	**(F) Ratio** **(D)/(E)**	**(G) Norm. Ratio** **(F)/2.35**
Range of Price								
|R|=15		2,293,360	3,369,742	0.68	1,340,000	1,121,414	1.19	0.51
|R|=31		4,428,144	5,097,334	0.87	2,620,000	1,405,418	1.86	0.79
|R|=63		8,697,712	7,157,309	1.21	5,180,000	1,423,967	3.63	1.55
|R|=127		17,236,848	9,549,645	1.80	10,300,000	1,636,989	6.29	2.68
|R|=255		34,315,120	12,274,426	2.80	20,540,000	2,121,623	9.68	4.12
|R|=511		68,471,664	15,331,680	4.47	41,020,000	2,161,902	18.97	8.07
|R|=1023		136,784,752	18,721,328	7.31	81,980,000	2,315,035	35.41	15.07
|R|=2047		273,410,928	22,443,544	12.18	163,900,000	2,329,521	70.36	29.94
|R|=4095		546,663,280	26,498,268	20.63	327,740,000	3,416,721	95.92	40.82

## Abbreviations

The following abbreviations are used in this manuscript:

DER	Distributed Energy Resources
DET	Distrubuted Energy Trading
DSO	Distributed System Operator
FE	Functional Encryption
FHIPE	Function-Hiding Inner Product Encryption
IEA	International Energy Agency
IoE	Internet-of-Energy
IPE	Inner Product Encryption
OID	One-time identifier
P2P	Peer-to-Peer
PPT	Probabilistic Polynomial Time
PUFs	Physical Unclonable Functions
RES	Renewable Energy Systems
UID	User identifier
UN	United Nations
